# Reintroducing *B. infantis* to the cesarean‐born neonate: an ecologically sound alternative to “vaginal seeding”

**DOI:** 10.1093/femsle/fnaa032

**Published:** 2020-02-18

**Authors:** Rebbeca M Duar, David Kyle, Rachel M Tribe

**Affiliations:** 1 Evolve BioSytems, Inc. Davis, California, 95618 USA; 2 Dept. of Women and Children's Health, School of Life Course Sciences, St Thomas' Hospital, London SE1 7EH UK

**Keywords:** mode of delivery, caesarean section, vaginal birth, probiotics, microbiome

## Abstract

There is a burgeoning literature highlighting differences in health outcomes between babies born vaginally and by caesarean section (c-section) This has led to the suggestion that infants born by c-section may benefit from vaginal swabbing/seeding. Here, we discuss from an ecological perspective that it is gut-adapted, not vagina-adapted microbes that are likely to take up residence in the gut and have the most beneficial impact on the developing neonate. Further, we caution the practice of ‘vaginal seeding’ may be potentially unsafe and also give parents and health professionals a false sense of action in restoring the infant gut microbiome following c-section. Instead, we argue that restoring *B. longum* subsp. *infantis*, which has evolved to colonize the infant gut, is a safe and ecologically-sound approach to restoring the gut microbiome of infants born by c-section.

## Teaser

Restoring the gut microbiome of infants delivered by cesarean section with *B. infantis*.

## EVIDENCE, RISKS AND ECOLOGICAL CONSIDERATIONS OF VAGINAL SEEDING

There is a large body of literature indicating that the long-term health outcomes of babies delivered by caesarian section (c-section) are different than those delivered vaginally. The largest single study of this kind included 1.9 million infants born between 1977 and 2012. The analysis showed significantly increased risks of asthma, juvenile arthritis, inflammatory bowel disease (IBD), immune deficiencies and leukemia in the c-section group compared to babies delivered vaginally over the same time period (Sevelsted *et al*. 2015). It is also well known that babies born by c-section have a gut microbiome that is different from babies born vaginally (Dominguez-Bello *et al*. 2010; Backhed *et al*. 2015; Shi *et al*. 2018; Shao *et al*. 2019), which has led to the assumption that it might be beneficial to inoculate c-section delivered infants with a swab of their mother's vaginal microbiome at the time of birth in an attempt to mimic the microbial exposures encountered through a vaginal delivery. Increased awareness of the importance of the microbiome in early life has sparked considerable interest amongst parents who wish to undertake ‘vaginal seeding’. This has been paralleled recently with an interest in vaginal microbiome transplantation between women (Lev-Sagie *et al*. 2019). However, the scientific and medical communities remain cautious on the safety and efficacy of these yet unproven clinical practices.

One main issue with ‘vaginal seeding’ is that the proposed link between vaginally associated microbes to the infant gut microbiome has limited ecological foundation. Similar to the many different terrestrial and marine ecosystems on our planet that support their own unique variety of flora and fauna, our bodies too have different ecosystems that also support their own unique species of specialized microbes (Lloyd-Price *et al*. 2017). Like the plant and animal communities in Earth's biomes, microbial communities cannot be transferred to a different biological niche and be expected to flourish. The community of bacteria colonizing the vaginal canal is significantly different from the microbial communities thriving on the skin or in the gut because the environmental conditions and nutritional niches of these communities are all vastly different. This is supported by the fact that while vaginal bacterial strains can be transiently found in the infant gut soon after birth, these species tend to be in low abundance and can be readily lost or replaced by gut-adapted species. Further, assuming vaginal species do provide a health benefit, maintaining stable populations in the infant gut would require constant re-inoculation, which could not be achieved via a one-time procedure of ‘vaginal seeding’. Conversely, strains originating from the maternal gut form stable populations in the infant gut microbiome (Ferretti *et al*. 2018; Wampach *et al*. 2018; Yassour *et al*. 2018). This places the question on the efficacy of inoculating the infant with a purely vaginal source and suggests that providing the infant with gut-adapted strains might be more efficacious in resolving gut dysbiosis.

Providing that a one-time transfer of vaginal microbiomes is unlikely to be sufficient, the expected best outcome from ‘vaginal seeding’ would be that some of the introduced microbes would transiently colonize. However, without adequate safety screening using validated protocols, the process of vaginal swabbing carries a risk of inadvertently transferring potential pathogens, including genital herpes, Group B *Streptococcus*, chlamydia, and gonorrhea from an unhealthy vaginal microbiota, as well as additional nosocomial pathogens typically found in a hospital environment, directly to the baby (Huynh, Palasanthiran and McMullan 2018). Due to the insufficient evidence to show a benefit from the transient colonization of vaginal microbes and the potential risks with this process, there have been a number of medical opinion articles recommending that health professionals abstain from this practice, including the American College of Obstetricians and Gynecologists and the Danish Society of Obstetrics and Gynaecology (Committee on Obstetric Practice 2017; Haahr *et al*. 2018; Stinson, Payne and Keelan 2018).

**Figure 1.**
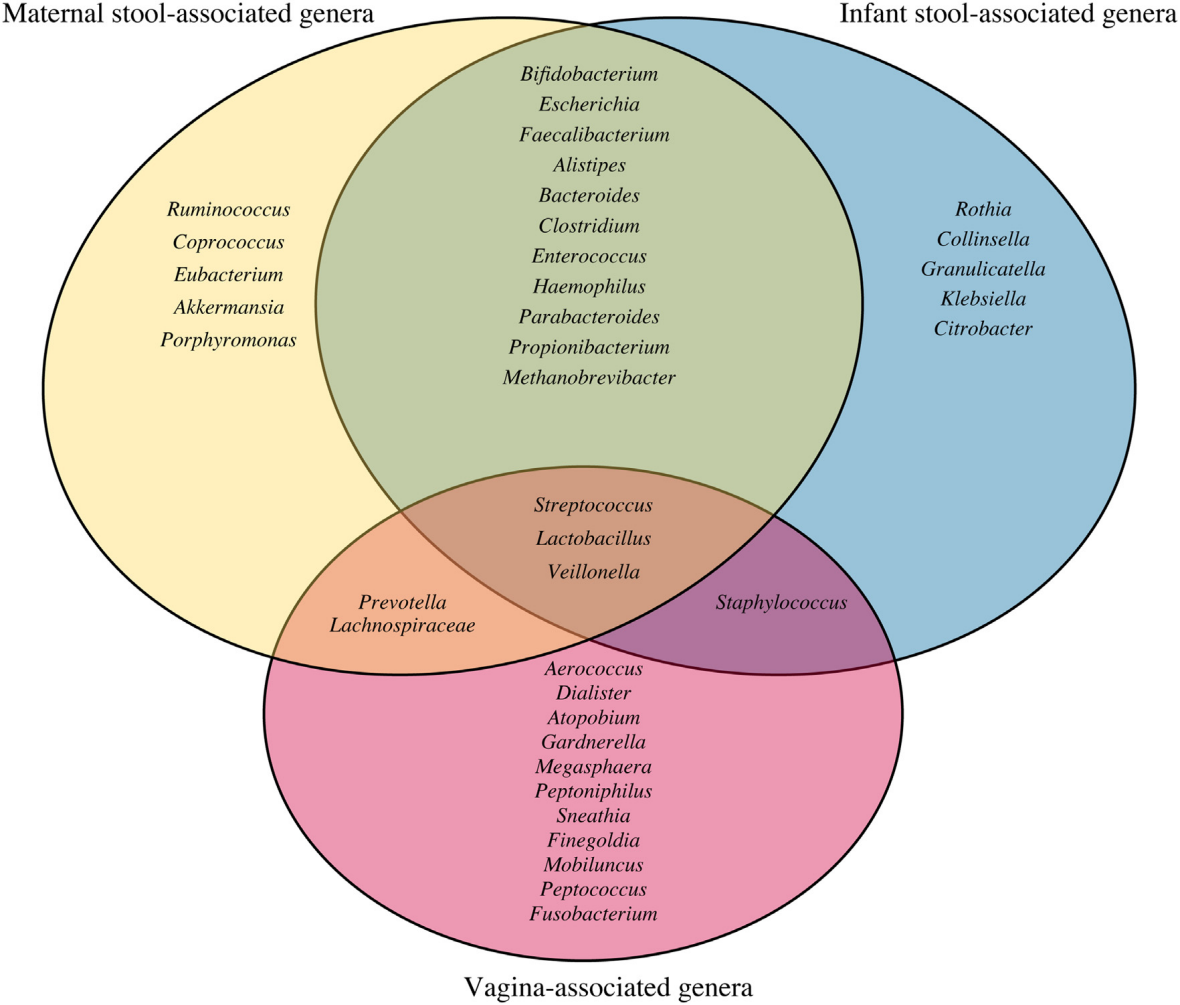
Venn diagram showing a substantial overlap between genera found in the stool of infants and maternal gut, but little overlap with genera found in the vagina. The list of gut-associated genera were obtained from studies characterizing the microbiome of stools collected during gestation (Koren *et al*. 2012; Goltsman *et al*. 2018) and after birth from mother and infant pairs (Asnicar *et al*. 2017; Ferretti *et al*. 2018; Goltsman *et al*. 2018; Wampach *et al*. 2018; Yassour *et al*. 2018). The list of vagina-associated genera were obtained from studies characterizing the vaginal microbiome during gestation (DiGiulio *et al*. 2015; Goltsman *et al*. 2018) and the postpartum period (MacIntyre *et al*. 2015) of women of diverse ancestries (Fettweis *et al*. 2019). Note that due to space constraints these lists are designed to be exemplary, not comprehensive. Only taxa for which representatives were detected in at least two independent studies were included.

When instead we consider the ecological conditions of the gut (anaerobic, highly competitive), the nutrients available (dietary and host derived glycans), and the birthing process itself, the most obvious seeding source of the infant gut microbiome is the gut microbiome of the mother. During a vaginal delivery, the baby's head presses on and flattens the mother's colon so that is very common for the mother's stool to be expelled to some extent. The proximity of the vagina and the anus and the optimal fetal position for birthing (occiput anterior position head down, facing towards the back of the mother's pelvis) facilitates the likelihood of maternal-infant microbial transfer (Trevathan 2015). Upon birth, the newborn infant gut has unoccupied ecological niches that are environmentally similar to the mother's gut, thus allowing vertically transferred bacteria from the mother's gut microbiome to colonize and thrive in this new space, in a quintessential fecal-oral transfer, as demonstrated in multiple studies tracking the transfer of microbes from mother to infant (Asnicar *et al*. 2017; Ferretti *et al*. 2018; Wampach *et al*. 2018; Yassour *et al*. 2018). In fact, the infant gut microbiome is dominated by taxa common in the maternal gut, while microbes associated with the vagina are much less prevalent (Fig. 1.) This pattern supports that the maternal gut microbiome represents the largest contributor of the infant microbiome.

## RESTORING THE MICROBIOME OF CAESAREAN SECTION BORN INFANTS WITH *B. INFANTIS*

Infancy represents a unique period when humans are evolutionarily adapted to thrive on a single source of nutrition (i.e. human milk). Thus, during this time, the breastfed infant gut microbiome is mainly shaped by the only glycans reaching the infant's colon, namely human milk oligosaccharides (HMO). The importance and role of these HMO is underscored by their abundance in human milk (15% of the energy content) and the inability of these compounds to be metabolized by the infant (Turroni *et al*. 2018). A small, select number of gut microbes can consume or metabolize these complex glycans in human milk. The most successful bacterium is *Bifidobacterium longum* subsp. *infantis* (*B. infantis*) as it has several unique gene clusters to capture, transport and internally metabolize all of the approximately 200 different HMO structures (Sela *et al*. 2008). This competitive advantage should make this bacterium the dominant microbe in the breastfed infant gut microbiome (Tannock *et al*. 2016), as is the case in many populations around the world where breastfeeding rates are high and microbiome-modifying interventions are less prevalent (Huda *et al*. 2019; Lawley *et al*. 2019). Unfortunately, this is not the case in most high income nations including the United States and New Zealand (Lewis and Mills 2017; Lawley *et al*. 2019). Historical references indicate that this change is a recent phenomenon (Henrick *et al*. 2018) that has coincided with the extensive use of antibiotics and the rise in planned, non-medically required c-section deliveries, especially in countries such as Brazil, where more than half of infants are born via elective c-section (Victora *et al*. 2011). This generational loss of *B. infantis* from the general population means that many babies born today are unlikely to naturally acquire *B. infantis* from their mothers even if born vaginally and breastfed (Tannock *et al*. 2016; Lawley *et al*. 2019) In a clinical study, the absence of *Bifidobacteriaceae* from a population of breastfed infants, was associated with an increase abundance of *Enterobacteriaceae*, *Clostridiaceae*, and *Bacteroidetes* (Frese *et al*. 2017). Few species in these families can metabolize HMO, but some are vigorous mucin degraders and there is now evidence of a significant erosion of the intestinal mucous layer in infants with low abundance of *Bifidobacteriaceae*, which is not observed in infants colonized with *B. infanti*s EVC001 (Karav, Casaburi and Frese 2018). Further, infants with low abundance of bifidobacteria and missing *B. infantis* have signatures of chronic enteric inflammation including elevated fecal TNFα, INFγ, IL1β, and calprotectin (Henrick *et al*. 2019), which have been linked to an increased prevalence of certain autoimmune disorders such as atopy and asthma later in life (Orivuori *et al*. 2015; Henrick *et al*. 2019). Overall, there is a repetitive trend between lower bifidobacteria in early life and a variety of chronic immune and inflammatory diseases, suggesting their presence in high abundance may have important roles in ensuring proper development during critical period of physical and immune maturation (O'Neill *et al*. 2017).

Feeding *B. infantis* EVC001 regardless of mode of delivery, robustly and persistently remodels the gut microbiome of infants and results in significantly increased levels of *Bifidobacterium* to up to 80% mean relative abundance of the total microbiome, as long as the infants are consuming human milk (Frese *et al*. 2017). These changes reflect the ecological adaptation of *B. infantis* to the infant gut and result in significant beneficial changes to the gut ecosystem (Duar *et al*. 2020), including increased production of organic acids that lower intestinal pH creating an environmental deterrent to the growth of potentially virulent enteropathogens (Casaburi and Frese 2018), antibiotic resistance taxa (Casaburi *et al*. 2019), and mucus eroding bacteria (Karav, Casaburi and Frese 2018). Altogether, these data suggest an evolutionary link between the mother (HMO from breast milk), the infant and *B. infantis* to synergistically create the ecological conditions required to protect the infant from potential infection and restore inflammatory markers to homeostatic levels (Henrick *et al*. 2019). These changes are durable and persist even after infants stop consuming *B. infantis*, which is in stark contrast to vaginal swabbing, which only partly and transiently alters the infant gut microbiome (Dominguez-Bello *et al*. 2016).

In conclusion, and viewed from an ecological and evolutionary perspective, it is gut-adapted, not vagina-adapted, microbes that are likely to take up residence in the infant gut (Fig. 1) and, thereby, have the most beneficial impact on the developing neonate. Thus, this and other ecological aspects of the infant gut presented in this opinion piece, should be considered when selecting probiotic strains and ingredients in infant formulas aimed to modulate the microbiome. Further, we and others (Cunnington *et al*. 2016; Haahr *et al*. 2018) concur that a continuation of the unsubstantiated practice of ‘vaginal seeding’ comes at a risk of not only being ineffective and potentially unsafe but gives parents and health professionals a false sense of action in restoring the infant gut microbiome following c-section delivery. Given the potential public health implications and economic burden associated with perturbed microbiome compositions (*i.e*. dysbiosis) in early life, we argue that scientific and medical focus should be placed instead on the rational design and implementation of strategies to reestablish the dominance of bifidobacteria in to the gut microbiome of infants, which shows the most promise in having a positive impact on life-long health outcomes (Duar *et al*. 2020). Restoring *B. infantis* to the infant gut microbiome represents one such approach that simply reestablishes the natural system of protection.
